# Angiotensin Receptor Blocker and Calcium Channel Blocker Preventing Atrial Fibrillation Recurrence in Patients with Hypertension and Atrial Fibrillation: A Meta-analysis

**DOI:** 10.1155/2021/6628469

**Published:** 2021-05-17

**Authors:** Haotian Ma, Hongcheng Jiang, Jing Feng, Yong Gan

**Affiliations:** ^1^The First Clinical School, Tongji Medical College, Huazhong University of Science and Technology, Wuhan, Hubei, China; ^2^Division of Cardiology, Department of Internal Medicine, Tongji Hospital, Tongji Medical College, Huazhong University of Science and Technology, Wuhan, Hubei, China; ^3^Department of Social Medicine and Health Management, School of Public Health, Tongji Medical College, Huazhong University of Science and Technology, Wuhan, Hubei, China

## Abstract

**Background:**

Atrial fibrillation (AF) is the most common serious cardiac rhythm disturbances and is responsible for substantial morbidity and mortality in general population. Hypertension is the most prevalent and potentially modifiable risk factor for AF. This study is aimed at evaluating the effect of angiotensin receptor blocker (ARB) or calcium channel blocker (CCB) on AF recurrence among patients with hypertension and AF.

**Methods:**

The PubMed, EMBASE, Medline, and Cochrane Collaboration of Controlled Clinical Trials registry databases were searched from their inception to September 2020.

**Results:**

A total of 7 randomized controlled trials (RCTs) enrolling 1495 patients were included in our study. This finding showed that ARB had a statistically significant superiority in preventing AF recurrence (OR: 0.43, 95% CI: 0.30-0.72, *P* = 0.0006) and persistent AF (OR: 0.41, 95% CI: 0.24-0.71, *P* = 0.001) compared to CCB. Subgroup analysis showed that there was a significant difference in telmisartan subgroup (OR: 0.54, 95% CI: 0.23-1.29, *P* = 0.17) and nontelmisartan subgroup (OR: 0.42, 95% CI: 0.23-0.77, *P* = 0.005). Subgroup analysis indicated that nifedipine subgroup did not show a statistically significant difference on AF recurrence between ARB and CCB (OR: 0.88, 95% CI: 0.46-1.68, *P* = 0.69), but amlodipine subgroup showed that ARB had a significant superiority in prevention of AF recurrence (OR: 0.39, 95% CI: 0.27-0.56, *P* < 0.0001) compared with CCB.

**Conclusions:**

This study suggests that ARB is superior to CCB for preventing the AF recurrence and persistent AF among patients with hypertension and AF.

## 1. Introduction

In patients with hypertension, atrial fibrillation (AF) is frequently observed and highly related with a series of fatal cardiovascular disease: heart failure, stroke, and myocardial infarction. Therefore, prevention and treatment of AF are urgently needed among these patients [1, 2]. Previous studies have shown that hypertension was the most common and potentially modifiable risk factor for AF [3–5], and antihypertensive treatment could reduce the risk of AF by reversing structural cardiac damage caused by hypertension [6, 7]. Though there are a variety of treatment for hypertension and AF, such as angiotensin receptor blocker (ARB) and calcium channel blocker (CCB), preventing structural changes may be an effect specific to ARB [8], which may prevent left AF, atrial fibrosis, dysfunction, and conduction velocity slowing [9]. This efficacy of ARB on AF has been confirmed in some studies [2, 10, 11]; however, others showed that there was a negative association [12, 13]. In addition, studies concerning lone CCB offered little experiment data, with researchers emphasizing merely on its antiarrythmia mechanism and side effects [14]. Thus, the prescription of ARB or CCB remains controversial. In major trials mentioned above, different types of antihypertensive medicine were prescribed in patients with some basic diseases, including hypertension, diabetes mellitus, and heart failure. These uncontrolled factors may affect the outcome estimation of the study. In order to evaluate a clearer magnitude of either ARB or CCB, this study is aimed at concentrating only on AF recurrence and persistent AF among patients with hypertension and AF.

## 2. Methods

### 2.1. Data Sources and Search Strategy

A meta-analysis was performed in accordance with standards set forth by the Preferred Reporting Items for Systematic Reviews and Meta-Analyses (PRISMA) statement [15]. The PubMed, EMBASE, Medline, and Cochrane Collaboration of Controlled Clinical Trials registry were searched using the key words “hypertension”, “atrial fibrillation”, “angiotensin receptor blocker”, and “calcium channel blocker”. Previous meta-analysis and other reviews related to the topic were reviewed to identify studies not included in this search strategy.

### 2.2. Inclusion Criteria and Exclusion Criteria

Studies meeting the following criteria were included in the meta-analysis: (1) the study design was RCT; (2) this study population was AF and hypertension patients; (3) the interest of exposure was ARB or CCB; (3) the interest of outcome was AF recurrence or persistent AF; and (5) the study reported the number of patients who had AF recurrence or persistent AF after treatment or provided sufficient information to allow their calculation.

Exclusion criteria were (1) patients included in the study had only atrial fibrillation and no hypertension or were not mentioned as having hypertension; (2) the drugs used in the study were not ARB compared with CCB; (3) the study only mentioned the incidence of atrial fibrillation, not the recurrence rate of AF or the rate of persistent AF; (4) studies were with duplication; (5) studies were ongoing or unpublished study, or the type of study was review and meta-analysis; (6) the follow-up of the studies was less than 30 days; and (7) studies were without access to full text for quality assessment or data extraction.

### 2.3. Data Extraction and Quality Assessment

Data were extracted in duplicate by two independent reviewers (HTM and HCJ), and any disagreements were resolved by consensus. The following information was extracted from the study: name of the author, year of publication, characteristics of study population at baseline, methods of exposure, outcome measurements, number of patients, and number of patients who had AF recurrence or persistent AF after treatment.

**Figure 1:**
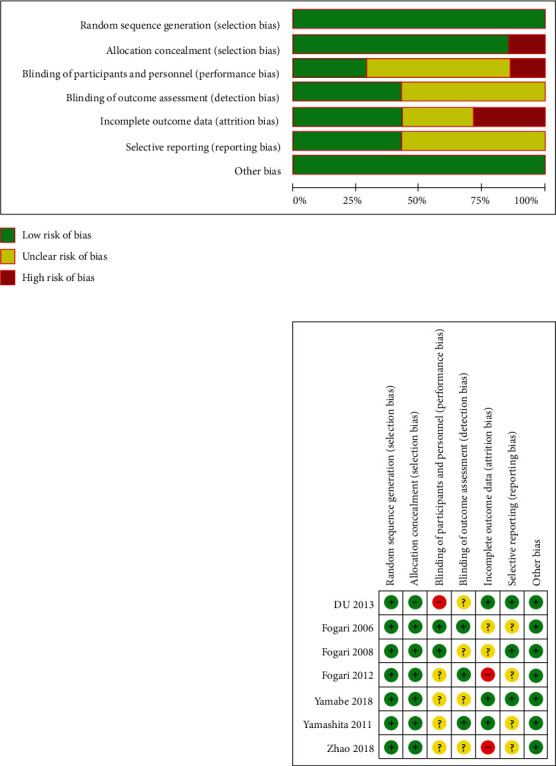
Risk bias assessment using the Cochrane risk of bias tools.

The methodological quality of each trial was evaluated for risk of bias using the standard criteria (Figure 1): random sequence generation; allocation concealment; blinding of participants, personnel, and outcome assessor; incomplete outcome ascertainment; selective reporting; and other potential sources of bias, which is recommended by the Cochrane Collaboration [16].

### 2.4. Data Synthesis and Statistical Analysis

Review manager 5.4 was applied to conduct all data synthesis and statistical analysis. The measured data were pooled in the study and analyzed using a random-effects meta-analysis model with inverse variance weighting. These were presented as odds ratios (ORs) with 95% confidence intervals (CIs). The magnitude of heterogeneity present was estimated using *I*^2^ statistics, and an estimate of the proportion of total observed variance attributed to the “between-study variance.” A random-effect meta-regression analysis was performed to identify potential effect modified factors. All *P* values were 2 tailed with the statistical significance set at .05.

## 3. Results

### 3.1. Study Selection and Evaluation

**Figure 2:**
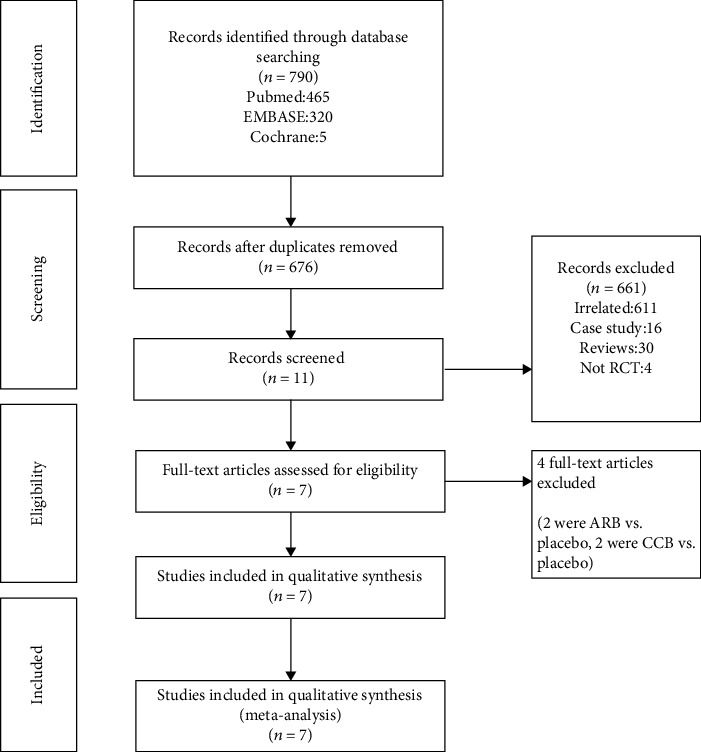
Flow charts showing relevant studies.

A flow chart showing the study selection is presented in Figure 2. We identified 790 potential articles from four electronic databases. After removing duplicates, 676 studies were screened by titles and abstracts. 661 studies were excluded because of noncompliance with the inclusion criteria. 11 studies were assessed by full articles for eligibility, and 4 articles were excluded for improper control. Finally, 7 studies were included in this meta-analysis.

### 3.2. Study Characteristics

The basic characteristics of seven studies are summarized in Table 1. The seven eligible studies included 1495 patients with hypertension and AF. Patients' age of included studies ranged from 55 to 75 years old. All patients in sinus rhythm had experienced an ECG-documented AF episodes in last 6 months. The follow-up of included studies ranged from 0.5 to 2 years; the median was 1 year. As to ARB category, patients of two studies were prescribed with telmisartan [8, 10], two studies with valsartan [11, 17], one study with losartan [18], one with irbesartan [19], and one with candesartan [20]. As to CCB category, patients of six studies were prescribed with amlodipine, [10, 11, 17–20] and one with nifedipine [8]. Two studies were conducted in China [8, 17], two in Japan [19, 20], and three in Italy [10, 11, 18].

### 3.3. The Effect of ARB and CCB on AF Recurrence and Persistent AF

**Figure 3:**
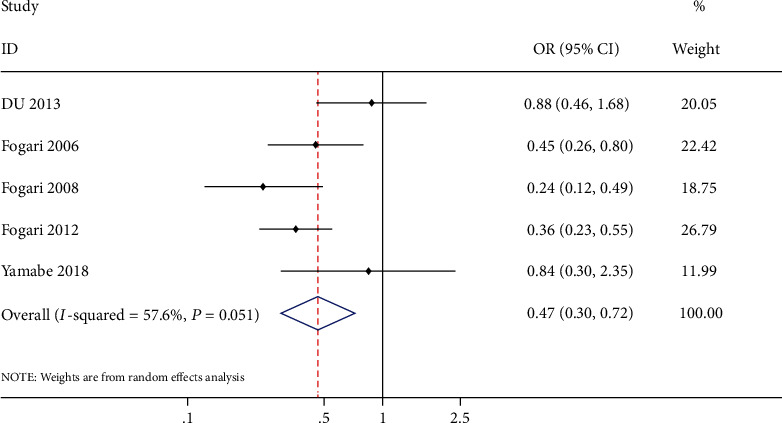
Forest plot of studies assessing the AF recurrence rate among patients with hypertension and AF.

**Figure 4:**
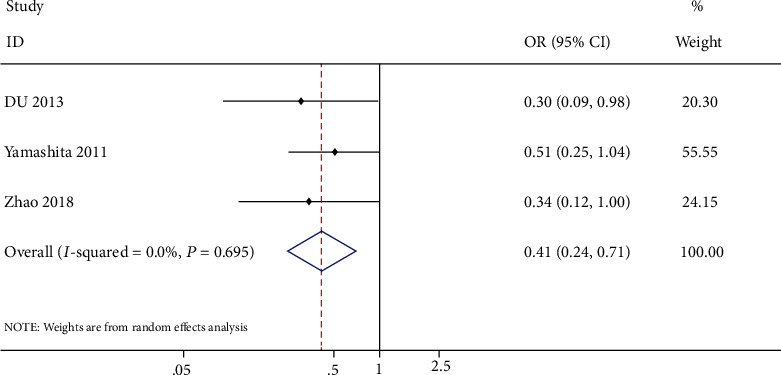
Forest plot of studies assessing the persistent AF rate among patients with hypertension and atrial fibrillation.

A total of 7 trials enrolling 1495 patients were included in this study [8, 10, 11, 17–20]. 744 patients were prescribed with ARB and 751 with CCB. This finding showed that ARB had a statistically significant superiority to CCB in preventing AF recurrence (OR: 0.47, 95% CI: 0.30-0.72, *P* = 0.0006, *I*^2^ = 57.6%) (Figure 3) and in preventing persistent AF (OR: 0.41, 95% CI: 0.24-0.71, *P* = 0.001, *I*^2^ = 0%) (Figure 4).

### 3.4. Subgroup Analysis concerning Telmisartan Group and Nontelmisartan Group

**Figure 5:**
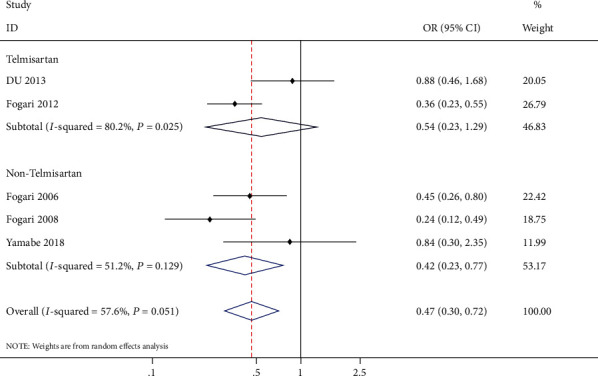
Forrest plot of subgroup analysis with the telmisartan group and nontelmisartan group in AF recurrence among patients with hypertension and AF.

Subgroup analysis was conducted to evaluate the telmisartan group and nontelmisartan group (Figure 5). The telmisartan subgroup enrolled two studies [8, 10], and there was no statistically significant difference between ARB and CCB (OR: 0.54, 95% CI: 0.23-1.29, *P* = 0.17), and significant statistical heterogeneity was found (*P* = 0.02, *I*^2^ = 80.0%). Whereas the nontelmisartan subgroup enrolled three studies [11, 18, 19] and compared with CCB, ARB had a statistically significant superiority in prevention of AF recurrence (OR: 0.42, 95% CI: 0.23-0.77, *P* = 0.005) with medium heterogeneity (*P* = 0.129, *I*^2^ = 51.2%).

### 3.5. Subgroup Analysis concerning Nifedipine Group and Amlodipine Group

**Figure 6:**
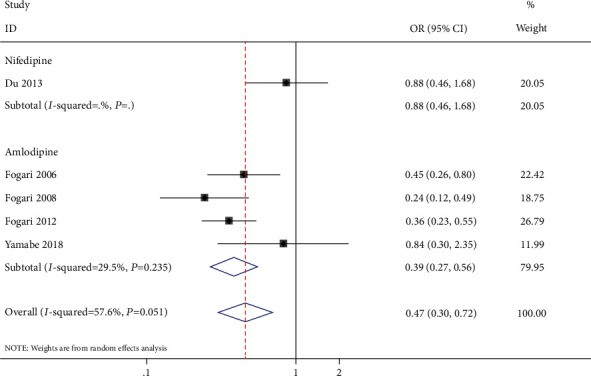
Forrest plot of subgroup analysis with the nifedipine group and amlodipine group in AF recurrence among patients with hypertension and AF.

Subgroup analysis was conducted to assess the nifedipine group and amlodipine group (Figure 6). Nifedipine was prescribed in only one study [8], and amlodipine was prescribed in four studies [10, 11, 18, 19]. In the nifedipine subgroup, there was no statistically significant difference between ARB and CCB (OR: 0.88, 95% CI: 0.46-1.68, *P* = 0.69). On the contrary, the amlodipine subgroup showed that ARB had a statistically significant superiority in prevention of AF recurrence (OR: 0.39, 95% CI: 0.27-0.56, *P* < 0.0001) with medium heterogeneity (*P* = 0.235, *I*^2^ = 29.5%) compared with CCB.

## 4. Discussion

The finding suggests that ARB shows statistically significant superiority to CCB in preventing AF recurrence and persistent AF.

ARB providing better prevention of AF recurrence could be interference with ion-channel function and modulation of refractoriness, inhibition of Ang II–induced fibrosis, reduced atrial stretch, improved left ventricular hemodynamics, and modulation of sympathetic nerve activity [21, 22]. Similar conclusions were also displayed in reviews below, as Kumagai stated that ARB can prevent structural remodeling [23], and Nakashima believed that ARB can prevent electrical remodeling induced by short-term rapid atrial pacing [24].

As comparison, studies concerning CCB alone offered little experiment data, with researchers merely emphasizing its antiarrythmia mechanism and side effects [14]. As mentioned before, antihypertensive treatment could reduce the risk of AF by reversing structural cardiac damage caused by hypertension [6, 7], but preventing structural changes may be an effect specific to ARB [8], which was not discovered yet in CCB.

Subgroup analysis was conducted to evaluate the telmisartan group and nontelmisartan group. Telmisartan was prescribed in two studies [8, 10], which might contribute to its AF-preventive effect through its insulin-sensitizing effect and the attenuation of AF-promoting atrial remodeling related to peroxisome proliferator-activated receptor gamma stimulation. In contrast, the other ARBs did not appear same potential for interaction with the receptor like telmisartan [25]. One study mentioned that AF recurrences rate was significantly lower in the telmisartan-treated patients than other antihypertensive drugs-treated patients who suffered hypertension with AF previously [26]. Our subgroup analysis showed significant differences between the telmisartan subgroup and the nontelmisartan subgroup, but only nontelmisartan ARB had a better effect on AF recurrence prevention. Further research is required to determine whether telmisartan is superior to other ARB in preventing AF recurrence and hypertension.

A negative outcome was observed in his study conducted by Du et al. [8]. A high heterogeneity (*P* = 0.051, *I*^2^ = 57.6%) was detected when this study was included, compared with much lower heterogeneity (*P* = 0.235, *I*^2^ = 29.5%) when this study was excluded. Inclusion and exclusion criteria, study methods, and other contents were compared to determine the origin. Based on available data, several possible causes were discovered with different CCB categories and different sex proportions.

Subgroup analysis was conducted to evaluate if different CCB categories were the origin of high heterogeneity. The result indicated that nifedipine may perform better in prevention of AF recurrence than amlodipine. Though one study mentioned that nifedipine could treat hypertension by inhibiting aldosterone release and further more reducing AF recurrence [27]. More studies confirmed that amlodipine leads to little reflex tachycardia and a lower incidence of vasodilator side effects when compared with nifedipine [28, 29]. Theoretically, amlodipine should carry out lower AF recurrence rate than nifedipine. Due to the contradictory conclusions, this difference in CCB category might contribute to high heterogeneity of the study.

Different sex proportion in studies could also be a possible factor for high heterogeneity. Proportion of male patients was 61.74% in Du 2013 study but 45.71% in Fogari 2008. Based on current studies, all sex differences in cardiovascular conditions have their basis in the combined expression of genetic and hormonal differences between women and men [30]. And women should be considered for higher sensitivity towards antihypertension and anti-AF treatment. However, exact sex proportion in the outcome was not displayed in any study; therefore, subgroup analysis could not be conducted. Further investigations and data were required to determine whether sex is a major impact on the outcome. Other than different CCB category and sex proportion, long history of hypertension may also affect the outcome. Fogari stated that the probability of eliminating AF completely is likely to be related to a point of no return of structural atrial remodeling [10]. The mean history of hypertension was about 9 years in Du's study, and according to this study's inclusion criteria, it was possible that patients enrolled did not go through proper treatment with hypertension in an early stage, causing more severe atrial structural remodeling than patients in other studies.

Above all, high risk of performance bias should also be taken into consideration in this study.

### 4.1. Strengths and Limitations

This is the first study showing effects of ARB and CCB in prevention of AF recurrence and persistent AF in patients with hypertension and AF, which may offer a better choice for doctors when they face a patient with hypertension and AF. Our study chose to concentrate only on AF patients with hypertension and AF, and patients with other diseases were excluded to eliminate influence as much as possible so that we could accurately comprehend the magnitude of both ARB and CCB. In addition, superiority of different ARB or CCB categories was evaluated in subgroup analysis to provide more information and suggestions. A major limitation of this study was the lack of adequate data. Not only did we fail to include many eligible articles but also the articles presented primary endpoints in various ways, which led to a small amount of data collected.

## 5. Conclusions

Our meta-analysis suggests that ARB had a statistically significant superiority to CCB in prevention of AF recurrence and persistent AF among patients with hypertension and AF. Given the increasing prevalence of hypertension worldwide, this finding may offer a practical and valuable clue for the prevention of AF recurrence.

## Figures and Tables

**Table 1:** Characteristics of studies included in the meta-analysis.

Study source	Follow-up (years)	Age at baseline (years)	No. of participants	Exposure assessment	ARB categories	CCB categories
Du et al., 2013 [8]	2	55-69	149	Questionnaire, 12-lead ECG, 24-hour Holter monitoring	Telmisartan	Nifedipine
Fogari et al., 2008 [11]	1	58-72	245	12-lead ECG, 24-hour Holter monitoring	Valsartan	Amlodipine
Fogari et al., 2006 [10]	1	56-71	222	12-lead ECG, 24-hour Holter monitoring	Losartan	Amlodipine
Fogari et al., 2012 [18]	1	60-75	378	12-lead ECG, 24-hour Holter monitoring	Telmisartan	Amlodipine
Yamabe et al., 2018 [19]	0.5	59-73	98	12-lead ECG, 24-hour Holter monitoring	Irbesartan	Amlodipine
Yamashita et al., 2011 [20]	1	57-75	318	12-lead ECG, 24-hour Holter monitoring	Candesartan	Amlodipine
Zhao et al., 2018 [17]	1	56-74	85	12-lead ECG, 24-hour Holter monitoring	Valsartan	Amlodipine

## Abbreviations

AF: Atrial fibrillation
ARB: Angiotensin receptor blocker
CCB: Calcium channel blocker.
